# Characterization of the transcriptional cellular response in midgut tissue of temephos-resistant *Aedes aegypti* larvae

**DOI:** 10.1186/s13071-025-06675-5

**Published:** 2025-05-14

**Authors:** Elisama Helvecio, Antonio Mauro Rezende, Maria J. R. Bezerra, Osvaldo Pompílio de-Melo-Neto, Maria Alice Varjal de Melo Santos, Tatiany Patrícia Romão, Constância Flávia Junqueira Ayres

**Affiliations:** 1 Instituto Aggeu Magalhães-FIOCRUZ, Av. Moraes Rego S/N Cidade Universitária, Recife, PE 50740-465 Brazil; 2 Instituto René Rachou-FIOCRUZ, Av. Augusto de Lima, 1715, Barro Preto, Belo Horizonte, MG 30190-002 Brazil

**Keywords:** *Aedes aegypti*, Temephos, Transcriptome, Metabolic resistance

## Abstract

**Background:**

Resistance to organophosphate compounds is a serious concern in dealing with the control of mosquito vectors. Understanding the genetic and molecular basis of resistance is important not only to create strategies aimed at detecting and monitoring resistance in the field but also to implement efficient control measures and support the development of new insecticides. Despite the extensive literature on insecticide resistance, the molecular basis of metabolic resistance is still poorly understood.

**Methods:**

To better understand the mechanisms of *Aedes aegypti* resistance to temephos, we performed high-throughput sequencing of RNA from the midgut tissue of *Aedes aegypti* larvae from a temephos-resistant laboratory colony, with long-term and continuous exposure to this insecticide (RecR), as well as from a reference, temephos-susceptible, colony (RecL). Bioinformatic analyses were then performed to assess the biological functions of differentially expressed genes, and the sequencing data were validated by quantitative reverse transcription-polymerase chain reaction (RT-qPCR).

**Results:**

The transcriptome analysis mapped 6.084 genes, of which 202 were considered upregulated in RecR, including known and new genes representing many detoxification enzyme families, such as cytochrome-P450 oxidative enzymes, glutathione-S-transferases and glucosyl transferases. Other upregulated genes were mainly involved in the cuticle, carbohydrates and lipid biosynthesis. For the downregulated profiles, we found 106 downregulated genes in the RecR colony, with molecules involved in protein synthesis, immunity and apoptosis process. Furthermore, we observed an enrichment of KEGG metabolic pathways related to resistance mechanisms. The results found in RT-qPCR confirm the findings of the transcriptome data.

**Conclusions:**

In this study, we investigated transcriptome-level changes maintained in a temephos-resistant *Ae. aegypti* colony under continuous and prolonged selection pressure. Our results indicate that metabolic resistance might involve a larger and more significant number of detoxification enzymes, with different functional roles, than previously shown with other mechanisms, also contributing to the resistance phenotype in the *Ae. aegypti* RecR colony.

**Graphical Abstract:**

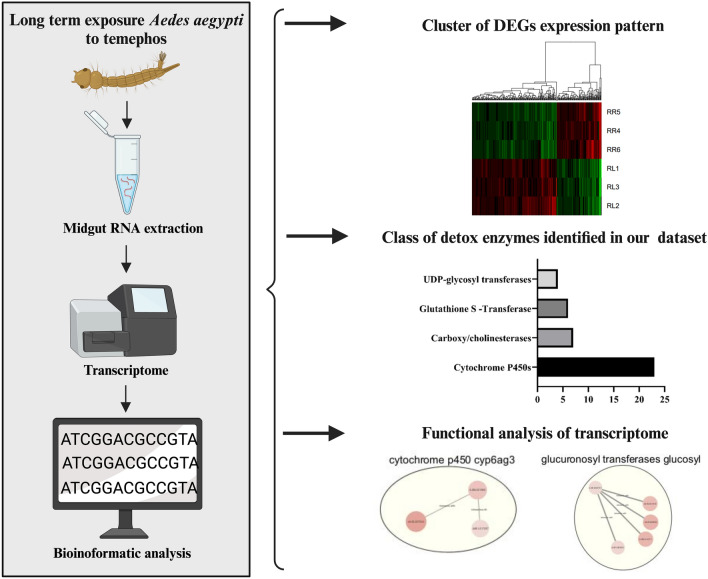

**Supplementary Information:**

The online version contains supplementary material available at 10.1186/s13071-025-06675-5.

## Background

*Aedes aegypti* is the main mosquito vector responsible for the transmission of pathogenic arboviruses, including those causing dengue fever, chikungunya and Zika, diseases affecting millions of people worldwide [1–4]. Some of the major strategies aiming the reduction of transmission of these diseases are therefore based on the control of vector populations [5–7]. In Brazil, the intensive use of chemical insecticides by the National Dengue Control Program (PNCD), for more than 2 decades, has favored the emergence of several resistant *Ae. aegypti* populations [8–10, 10–15]. These populations have developed the ability to survive even when exposed to doses of toxic compounds which would generally be lethal to non-treated populations [6, 17]. Resistance to chemical insecticides can affect the physiology, morphology and/or behavior of insects, [18–21] but does not occur uniformly in all species. It can arise due to the continued use and exposure to toxic compounds and be caused by the increase in prevalence of resistant alleles known to exist at a low frequency and carrying naturally occurring mutations [22].

The increase in insecticide biodegradation by detoxification enzymes, known as metabolic resistance, is one of the major molecular mechanisms related to chemical insecticide resistance. Elimination of toxic compounds occurs via chemical reactions which modify the xenobiotics in order to favor their excretion [18, 23, 24]. These can occur in three phases, with phase I consisting of reactions which cause these molecules to be more hydrophilic. Enzymes such as the multiple function oxidases (Cytochrome P450s or CYPs), which catalyze oxidation reactions, and the esterases, which catalyze the hydrolysis and breakdown of molecules containing ester groups, are involved [25–27]. The resulting compounds proceed to the second phase, where they will go through conjugation reactions mediated by transferases such as UDP glucoronosyl transferases, methyltransferases, sulfotransferases and glutathione-S-transferases (GSTs) [26, 27]. These reactions lessen their reactivity, toxicity and intracellular movement. Phase III, known as the export phase, employs efflux pumps that are responsible for removing the resulting compounds from the cell, with key participants being members of the transmembrane ABC carrier protein complex [28–31].

In previous studies, we used a laboratory-maintained *Ae. aegypti* colony selected for resistance to temephos (F_35_, RR_95_ ~ 225), named RecR [32], to investigate genetic and biochemical differences regarding the role of the GSTE2 enzyme in the metabolic resistance to this insecticide [33]. The resistant larvae displayed a substantially increased expression of the *GSTE2* transcript compared with individuals susceptible to temephos. Four unique missense mutations were detected in the *GSTE2 RecR* coding sequence, and the recombinant protein showed differences in catalytic activity against a synthetic substrate [33]. However, additional genes or gene families would need to be investigated to identify further expression patterns associated with the resistance phenotype and to improve its diagnosis. Other studies aimed at identifying multiple genes associated with resistance to chemical insecticides have more recently been performed based on the use of transcriptomic analyses, but these have primarily been conducted using mosquito field populations [34–40]. Using a transcriptomic approach to understand the complexity of the response to temephos in an *A. aegypti* colony continuously exposed to this larvicide in a controlled environment for over 20 years can further provide strong evidence for selection, whether positive or negative, of specific members of detoxifying enzyme families as well as other metabolic pathways associated with the resistance mechanism. This study hypothesizes that the continuous and prolonged exposure of the RecR lineage of *Ae. aegypti* to the insecticide temephos has selected specific genes from detoxification enzyme families, which are differentially expressed compared to the susceptible lineage RecLab.

This work then aimed to use RNAseq to do an expanded transcriptomic analysis of the gene expression profile and differentially regulated metabolic pathways in the RecR *Ae. aegypti* larvae. The characterization of differentially expressed genes in the RecR colony, compared to a susceptible reference colony (RecL), should contribute to understanding the metabolic resistance to temephos in this and other mosquito species. Indeed, the results from this study implicate known and new members of various families of detoxification enzymes to be probably associated with metabolic resistance to temephos in the *Ae. aegypti* RecR colony, with a consistent upregulation seen for multiple genes encoding enzymes belonging to multiple families of Cytochrome P450s (CYP) oxidases, as well as glutathione transferases, alpha-esterases, glutactin and UDP-glycosyl transferases. Other up-/downregulated genes in the resistant colony imply new mechanisms and a greater complexity associated with the resistance phenotype, a possible consequence of the longer period of continuous exposure to the insecticide.

## Methods

### *Aedes aegypti* strains

Two *Ae. aegypti* colonies were used in this study: (i) RecL, a susceptible colony originated from the municipality of Recife, PE; (ii) RecR, a laboratory-selected colony resistant to temephos, at generation F38 (RR95 ~ 250). Both colonies have been maintained for over 20 years at the Aggeu Magalhães Institute (IAM/Fiocruz-PE). Further details about the origin of these colonies have been previously described [32]. Both were kept at the insectary of IAM/ Fiocruz-PE, with larvae reared in dechlorinated tap water and fed with autoclaved cat food (Whiskas^®^), while the adults were maintained with a 10% sucrose solution and the females further fed with mouse blood. All larvae and adults were kept at 26 °C ± 2, 70% humidity and a 12 h light/12 h dark photoperiod.

### Collection and preparation of total RNA samples

For the transcriptomic analysis, 60 fourth instar larvae from both the RecR and RecL colonies were used. The midguts from these larvae were dissected and grouped into three pools of 20 midguts per colony, followed by total RNA extraction using the RNeasy Mini kit (Qiagen), according to the manufacturer’s instructions. The extracted RNA was then submitted to agarose gel electrophoresis to evaluate sample integrity and lack of DNA contamination. RNA purity and concentration were confirmed using a NanoDrop^®^ 2000 spectrophotometer (Thermo Scientific^®^) and the Qubit^™^ 2000 (Thermo Scientific^®^), respectively.

### Preparation of cDNA libraries, read quality check, trimming and mapping

The TruSeq Stranded mRNA Library Prep kit (Illumina) was used to prepare paired-end sequencing libraries with 2 µg from each total RNA sample (derived from 20 midguts), following the manufacturer's recommendations. Sequencing was performed using a MiSeq Reagent Kit V3^™^ (Illumina) for 150 cycles on an Illumina MiSeq Sequencer^™^ (Illumina) at IAM-FIOCRUZ. The quality of the sequencing reads was evaluated by applying the FastQC tool 0.11.5 (www.bioinformatics.babraham.ac.uk/projects/fastqc), followed by filtering and trimming using the Trimmomatic tool, version 0.36 [41], for the removal of low-quality sequences with average Phred < 30 and < 50 bp. Each library was then mapped against *Ae. aegypti* genome assembly, version AaegL5.1, from the Liverpool AGWG strain (LVP_AGWG) downloaded at 2019-02-14 from VectorBase (https://vectorbase.org/common/downloads/Legacy%20VectorBase%20Files/Aedes-aegypti/). Mapping and gene count reads were performed with RSEM (RNA-Seq by Expectation–Maximization), a software package that encapsulates the mapper and counts the reads by predicting the existence of isoforms from RNA-Seq data. The mapper used inside RSEM was Bowtie, version 1.3.2 [42], run with the recommended default settings.

### Differential expression and functional analyses

The R package DESeq2, version 1.40.2 [43], was used to perform the statistical analysis to identify the differentially expressed genes (DEGs). Biological replicates of the RecL and RecR colonies were compared, and only those genes that had at least five readings for all replicates under one of the two conditions were analyzed. Genes with Log_2_ Fold Change (LFC) absolute values ≥ 1.0 and with corrected *p*-value ≤ 0.05 were considered as DEGs. The scaled Heatmap was plotted using LFC with the Heatmap.2, from the gplots R package, and an MA plot was created where the DEGs were highlighted, using the “plot” function. DEGs identified as hypothetical proteins in the *Ae. aegypti* genome assembly were reassessed through additional annotation using the Blast2go tool, with default parameters, and searching through the Uniprot and NCBI (Blastnr) databases, version 5.2.5. Functional enrichment analysis using Gene Ontology (GO) terms was performed to identify GO terms significantly enriched (*p*-value ≤ 0.05) associated with DEGs (http://www.geneontology.org/). This analysis was performed for each of three GO ontologies (biological processes [BP], molecular functions [MF] and cellular components [CC]) using the STRINGdb R package, version 10.5. The pathway enrichment analysis of the Kyoto Encyclopedia of Genes and Genomes (KEGG) [44] was also performed using the STRINGdb R package [45]. The Pathview package implemented in the R programming environment was used to map DEGs, as well as their differential expression measurements, to the enriched KEGG pathways [46]. The DEGs were also visualized in protein networks present in the STRING database [45].

Protein interaction networks from STRING were downloaded as tabular files and visualized using Cytoscape, version 3.10.1 (www.cytoscape.org). To functionally annotate the network, the AutoAnnotate Cytoscape plug-in, version 1.2, was applied using the Adjacent Words algorithm (www.baderlab.org/Software/AutoAnotate). The colors and diameters of the network nodes change according to the LFC, with the intensity variation of the colors shifting towards blue indicating more negative LFC values and towards red indicating more positive LFC values. The thickness of the edges between nodes is determined by the combined score derived from STRING, serving as an indicator of confidence. All scores range from 0 to 1, with 1 representing the highest possible confidence. Several topological indices were calculated for each node using the Network Analyzer plugin. The layout applied was the edge-weighted Spring Embedded, using the combined score as the weight for the links between nodes. The node clusters were defined using the Markov Cluster algorithm, considering the combined score as the weight. Nodes connected by high-score links are likely to remain connected, while nodes connected by low-score links may lose their connection.

### Validation of RNAseq by quantitative real-time PCR (RT-qPCR)

Validation of gene expression observed in the RNAseq analyses was carried out by RT-qPCR targeting three upregulated (cytochrome P450 9J24-CYP9J24; cytochrome P450 12F6-CYP12F6; glutathione-S-transferase epsilon 2-GSTE2) and three downregulated genes (calreticulin–cal; hexaprenyldihydroxybenzoate methyltransferase–Methyl; metalloproteinase–Metallo). The primers were designed using the Primer Select program from DNAstar, version 17.1, based on the gene annotations available in VectorBase. The genes *actin* and *RPS17* were used as reference genes and are described in [47]. RT-qPCR was performed with the Quantitec SYBR Green RT-PCR kit (QIAGEN) applying specific primer pairs designed for each targeted gene (Supplementary Table 1). In this experiment, the RNA used was the same as used in the RNAseq experiment. All quantitative RT-PCR reactions were performed in triplicate on the Applied Biosystems real-time PCR system (ABI 7500). Each reaction used 3 μl of the RNA sample normalized to 100 ng, SYBR Green Master mix, 0.3 μM of each primer, 0.2 U of reverse transcriptase and PCR water to a final volume of 10 μl. The relative mRNA expression was calculated by the 2^–ΔΔCT^ method [48]. The RecL strain was used as a reference sample, and to assess differences in gene expression by RT-qPCR, statistically significant differences were determined with Student’s t-test using the GraphPad Prism software, version 8.

## Results

### RNAseq transcriptomic analyses of the RecR and RecL colonies

**Fig. 1:**
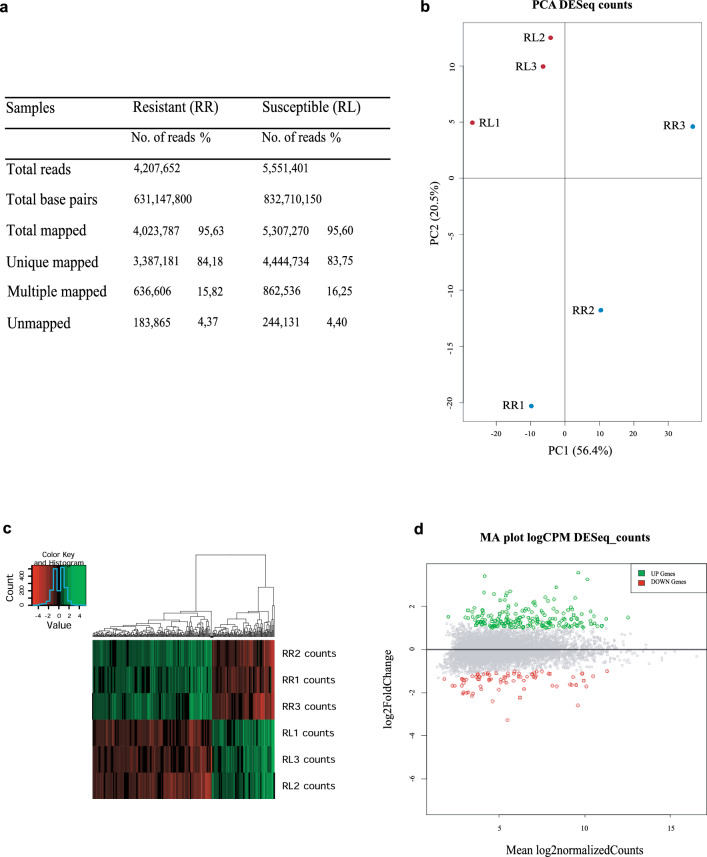
Differential expression analysis of RNAseq data. **a** Statistical analysis of mapping the RecR and RecL libraries' *Aedes aegypti* strains after quality control; **b** principal component analysis (PCA) of the RecL (RL1, RL2 and RL3, represented by red dots) and RecR (RR1, RR2 and RR3, represented by blue dots) replicates. **c** Heatmap of the RecR and RecL gene expression profile in *Ae. aegypti*. Upregulated genes are represented in green and downregulated genes in red. **d** MA plot showing the upregulated (green; cutoff > 1) and downregulated (red; cutoff < – 1) genes. All other genes (5775 in total, represented in gray) showed no significant differences in expression (*p* ≤ 0.05). In the MA plot, each dot represents a gene

Nearly 10 million reads were generated corresponding to six cDNA libraries derived from three biological replicates of the temephos-resistant RecR colony (RR1 to RR3) as well as three replicates from the susceptible RecL (RL1 to RL3 (Fig. 1a). Approximately 4.2 and 5.5 million reads were generated, respectively, for RecR and RecL, with average GC contents of 43 to 45%. After removal of low-quality sequences, roughly 96% of the reads were mapped against total transcripts of *Ae. aegypti* Liverpool strain with a unique mapping rate of ~ 84% for RecR and RecL for the mapped reads and a ~ 16% multiple mapping rate for both sets of replicates. A spontaneous scatter plot of the samples was next generated through a principal component analysis (PCA) of the reads. This analysis confirmed the formation of distinct groups for the biological replicates from each colony, with those from the susceptible colony clustering closer together, while those from RecR were more dispersed but showing profiles distinct from the RecL replicates (Fig. 1b). Considering a threshold minimum of five reads in each replicate of one experimental condition, 6084 genes were then mapped. As shown in the heatmap from Fig. 1c, each colony showed a unique pattern of gene expression, with distinct expression profiles consistently reproduced by their biological replicates. A total of 203 genes were considered upregulated for the RecR colony compared with RecL, with an absolute value of log_2_ fold change (LFC) ≥ 1, while 106 genes were downregulated and had LFC ≤ – 1, with corrected *p*-value ≤ 0.05. These genes are represented in the MA plot from Fig. 1d, with differentially expressed genes (DEGs) also listed in Supplementary Table 2. Many of these DEGs are defined as coding for uncharacterized proteins in VectorBase but most could be better defined following further additional searches through multiple databases. Their updated annotation is included in the supplementary table as well as the corresponding individual LFC and adjusted *p*-values. The large number of genes consistently found to be differentially expressed between the two colonies probably reflect multiple mechanisms that might differentially contribute to the metabolic resistance to temephos.

### Validation of differentially expressed genes through RT-qPCR

**Fig. 2:**
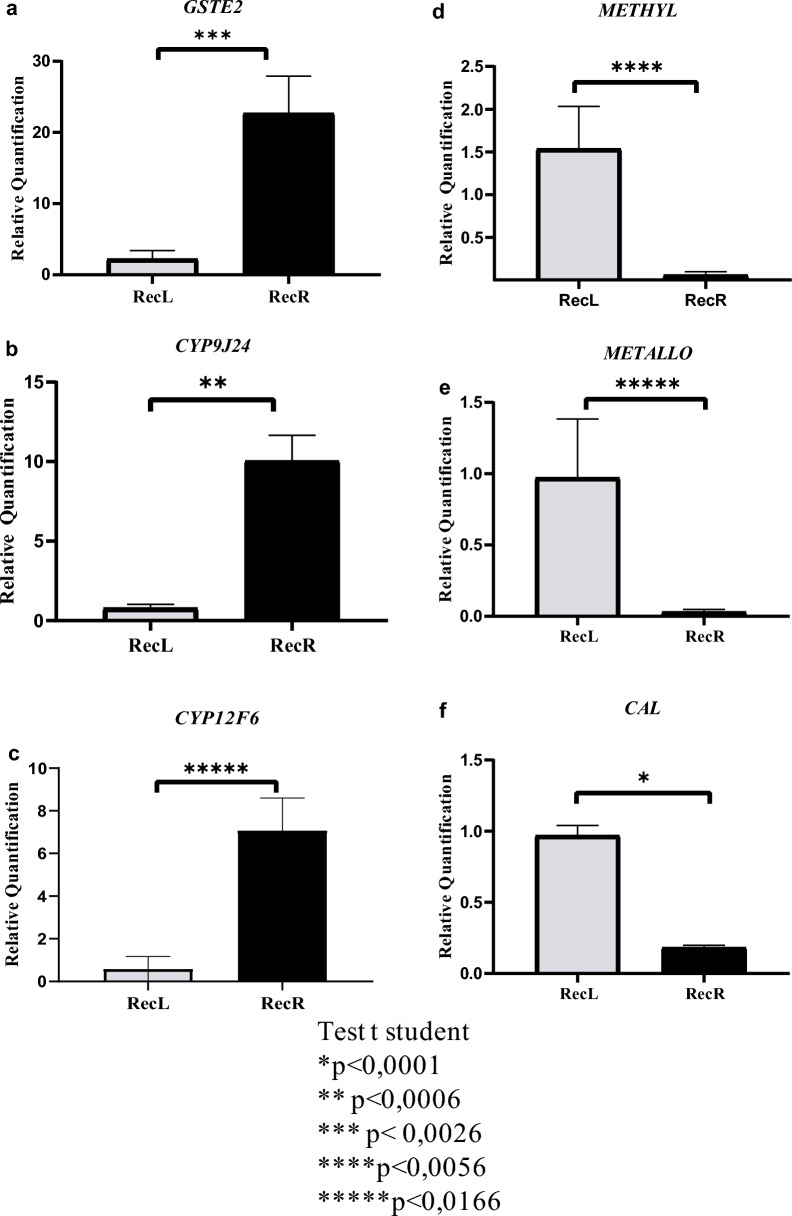
RT-qPCR assay for validating selected DEGs from RNAseq data. **a** Glutathione-S-transferase epsilon 2 (GSTE2); **b** cytochrome P450 9J24 (CYP9J24); **c** cytochrome P450 12F6 (CYP12F6); **d** calreticulin (Cal); **e** hexaprenyldihydroxybenzoate methyltransferase (Methyl); **f** metalloproteinase (Metallo). Gene expression levels are relative to those from the endogenous control genes, actin and RPS17, used for normalization. Means and standard errors were obtained from three biological replicates. *p < 0.0001, **p < 0.0006, ***p < 0.0026, ****p < 0.0056 and *****p < 0.0166

Validation of the RNAseq data was performed by selecting DEGs to evaluate their relative expression level through RT-qPCR, comparing the resistant and susceptible colonies, with the same samples submitted to RNAseq also used for this validation. The genes selected for this validation exhibited different relative expression values in RecR and/or were known to be relevant for the resistance to chemical insecticides. Transcripts encoding GSTE2 were first chosen because of this enzyme’s known biological relevance to the resistance event and to our previous studies specifically investigating its association with temephos resistance in the RecR colony [33, 49]. In the current analysis, the GSTE2 transcripts were mapped to two identical genes found in the most recent annotation to the *Ae. aegypti* genome at VectorBase. This leads to those transcripts being split into the two genes, with somewhat reduced LFC values found for each gene (LFC = 1.75 in Supplementary Table 2) but nevertheless showing increased abundance in RecR compared to the susceptible colony, as expected. Indeed, the relative expression of the GSTE2 transcripts was also significantly higher in RecR using the RT-qPCR evaluation, roughly tenfold higher than RecL (Fig. 2a), corroborating the RNAseq data. The gene encoding CYP9J24 was next chosen for the RT-qPCR analysis because it is the topmost upregulated gene in RecR (LFC = 3.55) and the RT-qPCR also revealed a major increase in its relative expression, roughly 14-fold higher than RecL (Fig. 2b). A similar result was seen for the CYP12F6 gene, the second topmost upregulated gene (LFC = 3.39), which also revealed a significant increase in its relative expression through RT-qPCR, ~ 12-fold more abundant in RecR compared to RecL (Fig. 2c).

To evaluate the downregulated transcripts in RecR, we arbitrarily selected three genes representative of different levels of downregulation. The gene encoding the enzyme hexaprenyldihydroxybenzoate methyltransferase (Methyl) was the second most downregulated gene in the RNAseq analysis (LFC = − 5.25), and indeed the RT-qPCR analysis showed a ~ 25-fold reduction in mRNA levels in the resistant colony (Fig. 2d). A second gene, encoding a metalloproteinase (Metallo) with lower levels of downregulation seen through the RNAseq (LFC = − 2.24), was found to be ~ 30-fold downregulated through RT-PCR (Fig. 2e), while the calreticulin gene (LFC = − 1.66) was ~ 5.4-fold less abundant in the resistant colony (Fig. 2f). A value of 0.88 was observed for the Pearson correlation coefficient comparing the relative expression levels obtained through RNAseq and RT-qPCR, confirming that the RT-qPCR differential expression profiles found for the selected genes are in agreement with and validate the corresponding profiles detected by RNAseq for these genes.

### Most relevant up- and downregulated genes in the RecR colony

Among the upregulated genes in RecR, 26 had LFC ≥ 2 and are listed in Table 1 for further analyses. Several of these belong to families related to metabolic resistance, with four multigene families found to be prominent and likely playing a significant role in the resistance to temephos in the RecR strain. Indeed, and considering the full range of upregulated genes from the Supplementary Table 2, 25 of those genes belong to different CYP clans or families of multiple function oxidases, including genes belonging to all four clans found in insects (CYP2, CYP3, CYP4 and mitochondrial clans). Seventeen of these upregulated genes belong to the CYP3 clan, from both CYP6 and CYP9 families, and are detailed in Table 2, with several of those, including five with LFCs ≥ 2 (CYP6AG3, CYP6AG7, CYP9J19, CYP9J24 and CYP9J28) [35, 50–56], previously shown to be upregulated in *Ae. aegypti* pyrethroid- and temephos-resistant populations [50, 52, 54–57]. Four of the remaining CYP genes belong to the mitochondrial clan, with one of those, the previously reported CYP12F6 gene [35, 55], being the second most upregulated gene and another, belonging to the CYP315 family (CYP315A1), a Halloween gene. The last three upregulated CYP genes belong to the CYP2 (CYP304B3) and CYP4 (three genes with lower LFC values) clans, with the CYP304B3 gene, also previously reported [54], found among the topmost genes.

**Table 1:** List of most up- and downregulated genes found for the temephos-resistant *Ae. aegypti* RecR colony

Upregulated genes				
No.	Gene ID	Name	LFC	*P-value_fdr*
1	AAEL014613	Cytochrome P450 (CYP9J24)	3.55	1.09E-49
2	AAEL002005	Cytochrome P450 (CYP12F6)	3.39	7.26E-03
3	AAEL006989	Cytochrome P450 (CYP6AG7)	3.24	1.19E-57
4	AAEL007024	Cytochrome P450 (CYP6AG3)	2.87	4.30E-48
5	AAEL025053	Clavesin 1	2.68	2.95E-19
6	AAEL014411	Cytochrome P450 (CYP304B3)	2.64	8.99E-11
7	AAEL002138	Triacylglycerol lipase putative	2.60	2.29E-07
8	AAEL027370	Long non-coding RNA (LncRNA)	2.56	9.13E-06
9	AAEL005179	Uncharacterized serine-rich protein	2.56	2.92E-13
10	AAEL002670	AMP dependent ligase	2.49	1.61E-06
11	AAEL021861	Cytochrome P450 (CYP6AG4)	2.41	3.50E-27
12	AAEL008560	Glucosyl/glucuronosyl transferases	2.38	1.80E-41
13	AAEL003132	Intraflagellar transport (ND5)	2.34	7.87E-05
14	AAEL014617	Cytochrome P450 (CYP9J28)	2.32	7.66E-24
15	AAEL021812	Uncharacterized protein	2.28	1.33E-13
16	AAEL011850	Cytochrome P450 (CYP315A1)	2.27	3.09E-12
17	AAEL014371	Glucosyl/glucuronosyl transferases	2.25	1.28E-29
18	AAEL027449	Long non-coding RNA (LncRNA)	2.23	6.26E-05
19	AAEL013262	Acidic endochitinase SP2	2.20	2.01E-25
20	AAEL023819	Long non-coding RNA (LncRNA)	2.19	2.68E-04
21	AAEL008266	endochitinase At2g43590-like	2.16	1.06E-04
22	AAEL028635	Cytochrome P450 (CYP9J19)	2.16	1.35E-15
23	AAEL022590	Kinesin-like protein subito isoform X2	2.12	1.46E-04
24	AAEL001816	Glucosyl/glucuronosyl transferases	2.07	2.92E-13
25	AAEL006990	Phospholipid phosphatase	2.03	1.23E-19
26	AAEL022132	Pancreatic triacylglycerol lipase	2.02	9.03E-12

Downregulated genes				
N**º**	Gene ID	Name	LFC	*P-value_fdr*
1	AAEL025999	40S ribosomal protein S17	− 7.23	2.38E-84
2	AAEL008330	Hexaprenyldihydroxybenzoate methyltransferase	− 5.25	2.51E-32
3	AAEL022291	Long non-coding RNA (LncRNA)	− 4.84	1.57E-26
4	AAEL015140	Protein NPC2 homolog	− 3.27	1.34E-26
5	AAEL025170	S-adenosylmethionine decarboxylase	− 3.24	3.70E-12
6	AAEL013004	Uncharacterized protein	− 2.63	2.39E-06
7	AAEL010963	Brain chitinase and chia	− 2.60	9.66E-49
8	AAEL001163	Macroglobulin/complement	− 2.55	7.35E-08
9	AAEL028017	Long non-coding RNA (LncRNA)	− 2.45	2.09E-07
10	AAEL027171	Uncharacterized protein	− 2.44	3.35E-05
11	AAEL001478	Bile acid beta-glucosidase. putative	− 2.40	7.85E-11
12	AAEL026761	Pseudogene	− 2.32	9.13E-05
13	AAEL011559	Metalloproteinase. putative	− 2.24	1.48E-04
14	AAEL024532	Toll-like receptor 6 isoform X2	− 2.24	7.80E-18
15	AAEL028195	Long non-coding RNA (LncRNA)	− 2.19	2.75E-04
16	AAEL021447	Galactosylgalactosylxylosylprotein 3- beta-glucuronosyltransferase	− 2.18	2.10E-05
17	AAEL006987	Putative phosphatidate phosphatase	− 2.17	1.10E-05
18	AAEL027453	Nucleoside diphosphate kinase	− 2.12	5.09E-04
19	AAEL022772	Long non-coding RNA (LncRNA)	− 2.08	1.65E-04
20	AAEL022345	Long non-coding RNA (LncRNA)	− 2.05	1.23E-04
21	AAEL024653	Protein son of sevenless	− 2.05	1.47E-05
22	AAEL009339	Activin receptor type I. putative	− 2.02	3.98E-05
23	AAEL002324	Vacuolar protein sorting-associated	− 2.02	6.43E-05
24	AAEL020314	Protein NPC2 homolog	− 2.01	1.72E-04
25	AAEL005073	Myosin-2 heavy chain-like	− 2.00	2.13E-06

Genes shown are those with log_2_ Fold Change (LFC) ≥ 2 or ≤ −2

**Table 2:** Data set analysis of differentially expressed detoxification genes in the RecR temephos-resistant strain classified by enzyme groups, class and families

Data based on *Aedes aegypti* Liverpool AGWG genome	Data based on the analysis of our transcriptome dataset				
Prominent multigenic families and total number of genes	Clan/class of detox enzymes and number of genes	Family of detox enzymes	Status of expression regulation	Genes	Log_2_ fold change
Cytochrome P450s (160 genes)	CYP2 (1)	CYP304	UP	CYP304B3 ◊	2.64
	CYP4 (3)	CYP4	UP	CYP4H31	1.38
			UP	CYP4D23	1.18
			UP	CYP4H33	1.01
	CYP3 (18)	CYP6	UP	CYP6AG7 + #	3.24
			UP	CYP6AG3 + ◊♣	2.87
			UP	CYP6AG4	2.41
			UP	CYP6BB2*	1.88
			UP	CYP6F3	1.59
			UP	CYP6AG4	1.51
			UP	CYP6M6	1.40
			UP	CYP6Z9	1.39
			UP	CYP6AA6	1.17
			UP	CYP6F2	1.16
			UP	CYP6M9*	1.09
		CYP9	UP	CYP9J24 +	3.55
			UP	CYP9J28 * +	2.32
			UP	CYP9J19*	2.16
			UP	CYP9M5*	1.85
			UP	CYP9J26	1.76
			UP	CYP9J6*	1.12
	Mitochondrial (3)	CYP12	UP	CYP12F6#♦	3.39
			UP	CYP12F5	1.78
		CYP315	UP	CYP315A1	2.27
Glutathione transferase (33 genes)	Epsilon (4)	–	UP	GSTE2	1.75
		–	UP	GSTE2	1.75
		–	UP	GSTE3	1.31
		–	UP	GSTE4*	1.16
	Sigma (1)	–	UP	GSTS1	1.05
	Theta (1)	–	UP	GSTT	1.1
Carboxy/cholinesterases (49 genes)	Alpha esterases (6)	–	UP	CCEae1C	1.51
			UP	CCEae3A†∞	1.47
			UP	Alpha esterase	1.31
			UP	CCEae3C	1.24
			DOWN	CCEae5C	− 1.67
			DOWN	Carboxylic ester hydrolase	− 1.67
	Glutactin (1)	–	UP	Carboxylic ester hydrolase	1.43
UDP-glycosyl transferases (32 genes)	UDP-glycosyl transferases (4)	UGT308	UP	Glucosyl/glucuronosyl transferases	2.38
		UGT308	UP	Glucosyl/glucuronosyl transferases	2.25
			UP	Glucosyl/glucuronosyl transferases	2.07
			UP	Glucosyl/glucuronosyl transferases	1.91

Reported upregulation in insecticide-resistant *Aedes aegypti* ◊[54]

+ [52]

^#^[55]

♣[56]

^*^[50]

♦[35]

^†^[51]

∞[53]

In addition to GSTE2 gene, we also identified further members of the glutathione-S-transferase (GST) epsilon class (GSTE3 and GSTE4) among the upregulated genes with LFC ≥ 1 and also genes belonging to the Sigma (GSTS1) and Theta (GSTT) classes (Table 2, Supplementary Table 2). Upregulated genes also include those encoding an enzyme belonging to the glutactin class (AAEL028198) as well as four alpha-esterases (CCEae1C, CCEae3A, CCEae3C and AAEL017071), all members of the multigenic family of carboxy/cholinesterases, with the CCEae3A gene also found previously to be upregulated in temephos-resistant mosquitoes [51, 53]. In addition, the UDP-glucosyl/glucuronosyl transferase family, belonging to the Phase II group of enzymes, active in the metabolism process of xenobiotics, was represented by four upregulated genes (Table 2, Supplementary Table 2). Further examples of upregulated genes encode proteins involved in DNA replication, recombination, repair and maintenance, as well as proteins involved in lipid and carbohydrate metabolism, intracellular transport, membrane vesicle formation, cellular motility and cell cycle regulation, and receptors involved with the detection of chemical and mechanical signals (Table 1, Supplementary Table 2).

Twenty-five genes on the list of downregulated genes in RecR were found with LFC ≤ – 2 and are shown in Table 1. The most downregulated gene encodes the 40S ribosomal protein S17. Another relevant downregulated transcript encodes the already mentioned hexaprenyldihydroxybenzoate methyltransferase, an enzyme that functions in the ubiquinone pathway and participates in ATP production. Protein products from the other genes listed in Table 1 and Supplementary Table 2 function in different biological processes, such as protein synthesis, apoptosis, digestion, immunity, hormone biosynthesis and others. At least two genes encoding for alpha-esterases, CCEae5C and esterase E4-like (carboxylic ester hydrolase), were also identified as downregulated but with a similar LFC ≤ – 1.67 (Supplementary Table 2).

Pseudogenes and genes encoding LncRNA (non-coding long RNAs) were also found within the list of differentially expressed genes. In the upregulated genes, two pseudogenes and seven LncRNA genes were found. Among these genes, three were found with LFC ≥ 2. For the downregulated genes, only one pseudogene was found, with LFC ≤ – 2, while nine LncRNA genes were also identified, with five of these with LFC ≤ – 2 (also listed in Table 1 and Supplementary Table 2). Overall, our results not only confirm previous reports implicating specific genes encoding metabolic enzymes with the resistance to temephos and related insecticides but also identify several new ones with likely direct roles associated with this resistance. The data also indicate that changes in several other biological processes, such as protein synthesis, might contribute further to the resistance mechanisms that need further investigation.

### Characterization of metabolic pathways and functional enrichment analysis

A functional enrichment analysis was carried out to identify metabolic pathways available in the KEGG database which are represented among the identified DEGs, providing an overview of the physiological processes involved with the resistance phenotype. As a result of this analysis, the DEGs were mapped to 75 metabolic pathways, with 42 of them considered significantly enriched, of which 37 had terms enriched with the upregulated genes and 5 were associated with the downregulated profile. Pathways with more genes found to be upregulated and with more significant *p*-values, shown in Table 3, include those associated with the metabolism of ascorbate and aldarate, sulfur metabolism and metabolism of galactose as well as a pathway related to glutathione metabolism and yet another pathway involved in valine, leucine and isoleucine degradation. Various other pathways are represented with lower *p*-values and by fewer upregulated genes and include several associated with the metabolism of different amino acids as well as pathways related to cytochrome xenobiotic or drug metabolism, fatty acid degradation and metabolism, insecticide hormone biosynthesis and others. In contrast, downregulated DEGs were found involved with protein processing in the endoplasmic reticulum and pathways related to the metabolism of nucleotides, vitamins and cofactors as well as the biosynthesis and metabolism of glycans (Table 4).

**Table 3:** Metabolic pathways mapped to upregulated genes in RecR

KEGG ID	Enriched KEGG terms	No. of genes	*P-value_fdr*
aag 00053	Ascorbate and aldarate metabolism	3	7.42E-04
aag 00920	Sulfur metabolism	3	6.93E-04
aag 00480	Glutathione metabolism	3	5.14E-04
aag 00280	Valine. leucine and isoleucine degradation	3	4.29E-04
aag 00052	Galactose metabolism	3	2.37E-04
aag 01212	Fatty acid metabolism	2	7.06E-03
aag 00330	Arginine and proline metabolism	2	5.92E-03
aag 00300	Lysine biosynthesis	2	5.69E-03
aag 00260	Glycine. serine and threonine metabolism	3	5.20E-03
aag 00071	Fatty acid degradation	2	4.39E-03
aag 01120	Microbial metabolism in diverse environments	2	3.71E-03
aag 00410	Beta-alanine metabolism	2	2.32E-03
aag 00640	Propanoate metabolism	2	2.32E-03
aag00860	Porphyrin and chlorophyll metabolism	2	2.32E-03
aag 00981	Insect hormone biosynthesis	2	1.60E-03
aag 00630	Glyoxylate and dicarboxylate metabolism	1	4.87E-02
aag 00380	Tryptophan metabolism	1	4.36E-02
aag00980	Metabolism of xenobiotics by cytochrome	1	4.26E-02
aag00982	Drug metabolism—cytochrome P450	1	4.26E-02
aag 00511	Other glycan degradation	1	4.26E-02
aag00062	Fatty acid elongation	1	4.18E-02
aag 00650	Butanoate metabolism	1	4.18E-02
aag 00790	Folate biosynthesis	1	4.18E-02
aag 02010	ABC transporters	1	4.07E-02
aag 00531	Glycosaminoglycan degradation	2	3.94E-02
aag 01040	Biosynthesis of unsaturated fatty acids	1	3.94E-02
aag 00604	Glycosphingolipid biosynthesis –	2	3.04E-02
aag 00230	Purine metabolism	1	3.04E-02
aag 00340	Histidine metabolism	2	3.04E-02
aag 00360	Phenylalanine metabolism	1	3.04E-02
aag 00450	Seleno compound metabolism	1	3.04E-02
aag 00592	Alpha-linolenic acid metabolism	1	3.04E-02
aag 04122	Sulfur relay system	1	3.04E-02
aag00750	Vitamin B6 metabolism	2	1.67E-02
aag 04142	Lysosome	2	1.65E-02
aag 04146	Peroxisome	1	1.52E-02

**Table 4:** Metabolic pathways mapped to downregulated genes in RecR

KEGG ID	KEGG enriched pathway	No. of genes	*P-value_fdr*
aag 04141	Protein processing in endoplasmic reticulum	5	1.87E-06
aag 01100	Metabolic pathways	6	8.54E-04
aag 00510	N-glycan biosynthesis	2	5.11E-03
aag 00740	Riboflavin metabolism	1	3.11E-02
aag 00232	Caffeine metabolism	1	1.67E-02

A functional enrichment analysis of up- and downregulated genes was also performed to identify over-represented Gene Ontology (GO) terms related to biological processes, molecular functions and cellular components. These were more associated with the upregulated genes, with 146 positively enriched terms identified, of which 46 were categorized in molecular function, 83 in biological processes and 17 in cellular components. Regarding the downregulated genes, 13 terms were identified, with 10 for molecular function and 3 for biological processes, with no enriched terms related to cellular components, all with *p*-value ≤ 0.05. These enriched GO terms grouped into functional categories are shown in the Supplementary Table 3 and Supplementary Table 4. As observed from the analyses of the individual genes differentially expressed between the two mosquito colonies investigated here, the investigation of the associated pathways and biological processes indicate that different metabolic pathways and other processes, which might not be considered directly related, might contribute to the resistance to temephos and enhance survival in the presence of the insecticide.

### Protein-protein interaction networks analysis

**Fig. 3:**
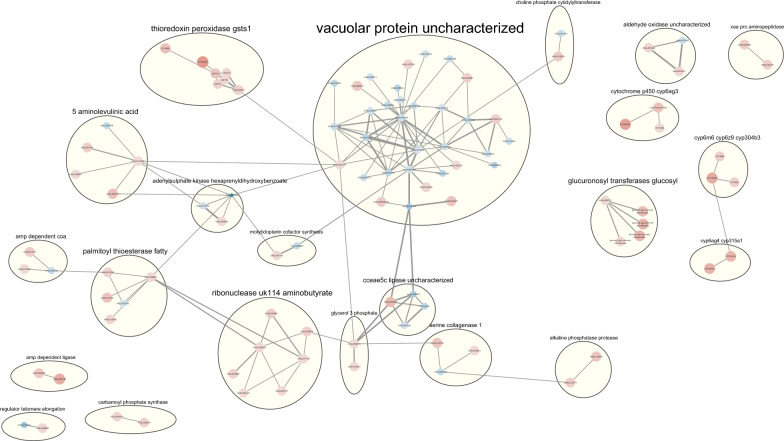
Protein-protein interaction networks are visualized using Cytoscape program. Functional protein-protein interaction networks of differentially expressed genes (DEGs) in the RecR strain. The colors and diameters of the nodes change according to the LFC, with the intensity variation of the colors shifting towards blue indicating more negative LFC values and towards red indicating more positive LFC values. The attributes of the nodes in the STRING interaction network are provided in Supplementary Table 5

Protein-protein interactions predicted according to the genes found to be differentially expressed were also investigated, with a total of 103 DEGs grouped into 22 clusters with various numbers of genes. These are detailed in Fig. 3 with a further list of the STRING clusters found for down- and upregulated genes, and the node attributes in the STRING interaction network, included in the Supplementary Table 5. Among these clusters, 13 were interconnected within a large group of interactions. The cluster with the highest number of genes by far was the one defined as “vacuolar protein uncharacterized,” with 33 genes, most of which were downregulated (24 genes). Proteins encoded by the genes in this cluster included a heat shock protein, the pyruvate dehydrogenase enzyme, a protein phosphatase, a late endosomal-lysosomal MP1 interacting protein, a homolog of vacuolar protein sorting-associated protein 28, transport and microtubule-associated proteins and others. Another cluster we considered relevant is named “thioredoxin peroxidase gsts1,” and it is organized with nodes comprising seven genes encoding proteins involved in the metabolism of xenobiotics and oxidoreductase activities, such as CYP6BB2, CYP6AG7, GSTE2, GSTE3, GSTS1, GSTE4 and thioredoxin peroxidase, all upregulated in the resistant colony. Several other small clusters were also found, including various comprising only genes encoding CYP proteins, some interlinked or not, with nearly all consisting mainly of proteins encoded by upregulated genes. These results highlight the prominent roles played by several of these clusters in the regulation of oxidation-reduction reactions and xenobiotic metabolic processes associated with the resistance event.

## Discussion

In Brazil, the organophosphate temephos was, for decades, the insecticide of first choice used by the *Ae. aegypti* control programs, with its indiscriminate use favoring the selection of resistant populations [58, 59]. This insecticide continues to be used in some Asian and American countries [60], although in Brazil it was replaced by others with a different mode of action in 2014 [59]. It was within this context, however, that the RecR colony was established for studies related to resistance to temephos [32], and it has since been maintained under selection pressure for > 40 generations. Our previous work using a microarray chip targeting 200 *Ae. aegypti* genes selected for possible involvement with metabolic resistance led to the identification of a small set of upregulated genes in the RecR colony [49]. The topmost two CYP genes found here to be upregulated in the RecR colony, belonging to the CYP6 and CYP9 families (CYP6AG7 and CYP9J24) were also found to be upregulated in the previous limited microarray analysis carried out previously. Other genes found to be upregulated in the previous analysis, and also identified here, are genes encoding several enzymes such as GSTE2 and GSTE3, the CCEae3A esterase, an aldehyde oxidase and the thioredoxin peroxidase. The microarray approach was limited in the number of genes investigated, but it does confirm the consistent upregulation of the cited genes, reinforcing the validity of the current transcriptomic results and the identification of new genes and pathways that could be involved with the resistance phenotype.

The analysis described here is consistent with other transcriptomic studies carried out with different species of insecticide-resistant mosquitoes and using related approaches [50–53, 61–67]. In *Ae. aegypti*, several studies have investigated resistance to pyrethroids as well as temephos [19, 50, 52, 61, 62, 64, 65]. As shown by our data, some of the most relevant results are related to the upregulation of different sets of CYP genes. Indeed, genes belonging to the CYP3 clan, which include both CYP6 and CYP9 families [68, 69], were readily found to be associated with insecticide resistance and xenobiotic metabolism early on [18, 19, 57, 64, 69–71]. The CYP6 family, the most represented in the CYP3 clan, is specific to insects and has evolutionary relationships with the vertebrates CYP3 and CYP5 families. Our results reinforce a role for specific CYP6 and CYP9 proteins, several not previously described, in mediating metabolic resistance to temephos and which also might induce cross-resistance to other relevant insecticides. Other upregulated CYP genes found here belong to the CYP2 and CYP4 clans, which nevertheless are known to have roles in development of sensory organs and odor production, respectively [68, 69]. Our results also evidenced a possible role for the gene encoding CYP315A1, a Halloween gene, in insecticide resistance, and since Halloween genes are known to encode enzymes that mediate the synthesis of ecdysteroids, insect-molting hormones that control the periodic molts of growing insects [72–74], this might reflect changes in insect development associated with insecticide resistance. The molecular mechanisms associated with the resistance mediated by the CYP genes still need to be better defined, but a study investigating the transcriptome of *Anopheles funestus* resistant to pyrethroids identified a cis-regulatory polymorphism in the CYP6P9A gene associated with its overexpression and reduction in effectiveness of insecticide-treated mosquito nets [75]. Closely related homologs to the CYP6P9A gene on the *Ae. aegypti* genome were identified as upregulated in our RNAseq data, but whether similar mechanisms are associated with their upregulation remains to be seen; a study on this would require sequencing on non-transcribed genomic regions of the resistant mosquitos.

Our study also identified a smaller number of upregulated genes encoding different GST enzymes. The Epsilon class GSTs, specifically GSTE2, have been frequently studied in African populations of *An. funestus* and *Anopheles gambiae* and blamed for resistance to DDT [76, 77]. GSTE2 has also been identified as important for the metabolism of insecticides in *Ae. aegypti* resistant to pyrethroids, DDT and temephos [62, 78, 79]. In our previous analysis, the upregulation of GSTE2 found for the RecR colony was associated with a RecR-specific allele [33]. These results are then in agreement with GSTE2 being the topmost upregulated GST observed here. Other GSTs from the same class, for instance, GSTE4 and GSTE7, were also reported to be upregulated in different studies [50, 62], consistent with GSTE4 also being found upregulated here. In our data, however, we did not identify any member of the Delta class in the differentially expressed genes, contrasting with the observation in *Anopheles sinensis* where this GST class was strongly associated with resistance to deltamethrin [66]. More detailed analyses of possible mutations in the coding, or even non-coding, regions of the upregulated GST genes might reveal further aspects restricted to the resistant individuals, but these analyses are beyond the scope of the present investigation and might be considered for further studies.

Several early studies also indicated that the esterase family of enzymes participate in organophosphate metabolism in *Ae. aegypti* [8, 13, 80, 81]. A subsequent analysis found the CCEae3A transcript to be upregulated in mosquito populations resistant to temephos and deltamethrin [62], and this was followed by gene expression analysis from the total transcriptome of mosquito populations resistant to temephos, *Aedes albopictus* from Greece and *Ae. aegypti* from Thailand, where both CCEae3A and CCEae6A genes were specifically upregulated [51, 53]. Considering the RecR colony, a QTL linked to resistance to temephos, on chromosome 2, was found associated with multiple esterase genes, including three confirmed to be upregulated here, encoding CCEae3A, CCEae1C and CCEae3C [82]. Our results are then consistent with multiple esterase genes being upregulated in RecR and likely other mosquito populations resistant to temephos, with CCEae3A being reproducibly upregulated in different studies. One unexpected finding was our observation that the CCEae5C gene was downregulated in the RecR colony, contrasting with a previous study where this gene was found to be upregulated in *Ae. aegypti* resistant to permethrin [83]. Specific functional assays might be considered in the future targeting the various enzymes identified here, coupled with further genomic sequencing and bioinformatic analyses, to better define their role in contributing to insect resistance to temephos.

Our results are consistent with proteomic analyses of *Ae. aegypti* strains resistant to chemical insecticides. We identified ten upregulated genes in the RecR strain that encode CYP enzymes (*CYP6AG4, CYP6BB2, CYP6AA6, CYP6M9, CCYPJ28, CYPJ19, CYP9M5, CYP9J26, CYP6J6, CYP315A1*), three GSTs (*GSTE2, GSTE3, GSTE4*) and one esterase (*CCEae3A*), previously reported with increased protein expression [39, 84, 85]. This demonstrates that resistance mediated by these enzymes in *Ae. aegypti* is associated with increased expression at both the transcriptional and protein levels. Studies on the protein expression of genes involved in temephos metabolism in *Ae. aegypti* are still limited. Therefore, the genes identified in this work require further investigation with functional genomics and proteomic approaches to be used as molecular markers in monitoring resistance to the insecticide temephos in the field.

Our transcriptomic analysis also led to the identification of various differentially expressed genes encoding proteases, kinases and phosphatases, as well as non-coding RNAs (LncRNAs), whose relevance for the resistance phenotype still needs to be better defined. LncRNAs, for instance, can regulate gene expression at chromosomal, transcriptional and post-transcriptional levels, playing a role throughout the entire process of cellular development [86, 87]. In insects, they have been associated with the post-transcriptional regulation of genes involved in various mechanisms of pesticide resistance [87–91], but the regulatory mechanisms by which they may be involved in *Ae. aegypti* remain unknown. Our protein-protein interaction analysis data are nevertheless consistent with the identified clusters of CYPs, GSTs and esterases serving as central orchestrators in cellular defense against xenobiotics [92]. These results were reinforced by the classification of genes differentially expressed in the Rec colony using Gene Ontology (GO) terms, which also highlight the functional relevance of molecular function categories associated with oxidoreductase and hydrolase activities, which were also significantly over-represented in *Culex quinquefasciatus*-resistant to permethrin [93]. Our findings thus show that although structurally different, organophosphate and carbamates insecticides induce common responses in terms of metabolic activity. Terms related to processes involved with DNA integrity, also found to be relevant, might further indicate that resistant larvae have a distinct pattern of response to DNA damage.

The most representative category obtained in our KEGG analyses was related to metabolism, with emphasis on the metabolism of ascorbate, glutathione, carbohydrates, amino acids and lipids, which may play an important role in mosquito fitness. In larval midgut tissue, temporal exposure to pesticides results in oxidative stress, which alters the metabolism of ascorbate and glutathione [94–96]. Both glutathione and ascorbate are abundant and stable antioxidants that interact with many different substances and pathways while maintaining a typically reduced state [97]. Numerous earlier findings indicate that glutathione depletion modifies the glycogen metabolism in order to control ascorbate production [98]. The existence of a biological cost for maintaining resistance to temephos in mosquitoes from the RecR colony has also been implied, and they are associated with a lower concentration of lipids and carbohydrates compared to susceptible individuals [99]. Overexpressed genes involved in lipid metabolism have also been observed in *Cx. quinquefasciatus* larvae resistant to *Lysinibacillus sphaericus* [100], while the concentration of the amino acid arginine has been shown to increase in *Cx. quinquefasciatus* exposed to temephos [101]. In our data, a pathway related to the metabolism of arginine and seven other amino acids was also significantly represented in the RecR colony. It highlights yet another relevant pathway which might also be associated with resistance to temephos and which might require a further investigation beyond the scope of the current study.

## Conclusions

The upregulation detected here for several new members of cytochrome P450 families, in addition to the upregulation of various known and new members of the carboxy/cholinesterase, glutathione transferase and UDP-glycosyl transferase families, indicates that a larger and more significant number of detoxification enzymes, with different functional roles, may potentially play key roles in the metabolic changes associated with temephos resistance in the *Aedes aegypti* RecR colony. Our results also imply that other mechanisms, such as the selective regulation of protein synthesis, possibly involving LncRNAs, might also be involved in the resistance to temephos. Overall, they reinforce the importance of further studies investigating the molecular mechanisms associated with the resistance event, helping define those that are most relevant and the chronology of their appearance during continuous exposure to the insecticide.

## Supplementary Information

Supplementary Material 1

## Data Availability

The transcriptome dataset is available on the NCBI platform with access code PRJNA1111491.
