# Supplementary material for: Characterization of the transcriptional cellular response in midgut tissue of temephos-resistant Aedes aegypti larvae
Source: Parasit Vectors. 2025 May 14;18:174. doi: 10.1186/s13071-025-06675-5 (PMC12076995; doi:10.1186/s13071-025-06675-5)
Supplement: Supplementary file 1 — Supplementary Material 1 [file 13071_2025_6675_MOESM1_ESM.zip › Supplementary table 1 Helvecio et al V9.docx]

**Supplementary table 1.** Specific primers used to validate the differential expression of selected genes by RT-qPCR.

| Gene | Primers (5’-3’) | Amplicon (bp) |
| --- | --- | --- |
| AAEL011197 (Actin) ^1^ | Forward CGTTCGTGACATCAAGGAAA  Reverse GAACGATGGCTGGAAGAGAG | 175 |
| AAEL004175 (RPS17) ^1^ | Forward AAGAAGTGGCCATCATTCCA  Reverse GGTCTCCGGGTCGACTTC | 200 |
| AAEL014613 (CYP9J24) | Forward CAAGCAGGTCGGCGTCAAAG  Reverse CTTCCAGCGCGAACCC | 130 |
| AAEL002005 (CYP12F6) | Forward GTGTTCAAAGGTCTGGGAG  Reverse CATCCCGGATCTTTAGC | 169 |
| AAEL007951 (GSTE2) | Forward AAGATCTACGGCTGGCTGGA  Reverse TCTGCGACAGGACAAACTGC | 100 |
| AAEL008330 (Metil) | Forward GCAAAACGGAGATCGAACACC  Reverse GTCTTGTTTTGTGATGTCCGCC | 164 |
| AAEL011559 (Metallo) | Forward CCGAGACCTTGACACCTG  Reverse CTCCTTCTAACATGCACTCAC | 210 |
| AAEL001005 (Cal) | Forward CGCGTCGGCCAAGTTC  Reverse GGTCCTTCTGGTCCACC | 137 |

^1^ Endogenous gene [47]. RPS17 (S17 ribosomal protein); CYP9J24 (Cytochrome P450 9J24); CYP12F6 (Cytochrome P450 12F6); GSTE2 (Glutathione-S-Epsilon transferase 2); Methyl (hexaprenyldihydroxybenzoate methyltransferase); Metallo (Metalloproteinase); Cal (Calreticulin).
