# Supplementary material for: Characterization of the transcriptional cellular response in midgut tissue of temephos-resistant Aedes aegypti larvae
Source: Parasit Vectors. 2025 May 14;18:174. doi: 10.1186/s13071-025-06675-5 (PMC12076995; doi:10.1186/s13071-025-06675-5)
Supplement: Supplementary file 1 — Supplementary Material 1 [file 13071_2025_6675_MOESM1_ESM.zip › Supplementary table 3 Helvecio et al V9.docx]

**Supplementary table 3.** Gene Ontology of enriched terms upregulated in the RecR colony.

| **Molecular Function** | | | | |
| --- | --- | --- | --- | --- |
| **Term description** | **Term identity** | **Proteins** | **Hits** | ***P-value_fdr*** |
| catalytic activity | GO:0003824 | 78 | 2 | 0.001080 |
| molecular_function | GO:0003674 | 164 | 2 | 0.001725 |
| farnesol dehydrogenase activity | GO:0047886 | 1 | 1 | 0.001725 |
| oxidoreductase activity. acting on the CH-OH group of donors. NAD or NADP as acceptor | GO:0016616 | 2 | 1 | 0.001725 |
| DNA helicase activity | GO:0003678 | 3 | 1 | 0.001725 |
| ATP-dependent DNA helicase activity | GO:0004003 | 3 | 1 | 0.001725 |
| ATP-dependent helicase activity | GO:0008026 | 3 | 1 | 0.001725 |
| DNA-dependent ATPase activity | GO:0008094 | 3 | 1 | 0.001725 |
| oxidoreductase activity. acting on CH-OH group of donors | GO:0016614 | 3 | 1 | 0.001725 |
| purine NTP-dependent helicase activity | GO:0070035 | 3 | 1 | 0.001725 |
| helicase activity | GO:0004386 | 4 | 1 | 0.002091 |
| ATPase activity. coupled | GO:0042623 | 6 | 1 | 0.002515 |
| 4 iron. 4 sulfur cluster binding | GO:0051539 | 6 | 1 | 0.002515 |
| ATPase activity | GO:0016887 | 7 | 1 | 0.002515 |
| iron-sulfur cluster binding | GO:0051536 | 7 | 1 | 0.002515 |
| metal cluster binding | GO:0051540 | 7 | 1 | 0.002515 |
| DNA binding | GO:0003677 | 9 | 1 | 0.003044 |
| oxidoreductase activity | GO:0016491 | 10 | 1 | 0.003136 |
| nucleoside-triphosphatase activity | GO:0017111 | 11 | 1 | 0.003136 |
| pyrophosphatase activity | GO:0016462 | 12 | 1 | 0.003136 |
| hydrolase activity. acting on acid anhydrides | GO:0016817 | 12 | 1 | 0.003136 |
| hydrolase activity. acting on acid anhydrides. in phosphorus-containing anhydrides | GO:0016818 | 12 | 1 | 0.003136 |
| hydrolase activity | GO:0016787 | 26 | 1 | 0.005417 |
| ATP binding | GO:0005524 | 28 | 1 | 0.005417 |
| adenyl nucleotide binding | GO:0030554 | 28 | 1 | 0.005417 |
| adenyl ribonucleotide binding | GO:0032559 | 28 | 1 | 0.005417 |
| nucleoside binding | GO:0001882 | 33 | 1 | 0.005417 |
| purine nucleoside binding | GO:0001883 | 33 | 1 | 0.005417 |
| purine nucleotide binding | GO:0017076 | 33 | 1 | 0.005417 |
| ribonucleoside binding | GO:0032549 | 33 | 1 | 0.005417 |
| purine ribonucleoside binding | GO:0032550 | 33 | 1 | 0.005417 |
| ribonucleotide binding | GO:0032553 | 33 | 1 | 0.005417 |
| purine ribonucleotide binding | GO:0032555 | 33 | 1 | 0.005417 |
| purine ribonucleoside triphosphate binding | GO:0035639 | 33 | 1 | 0.005417 |
| carbohydrate derivative binding | GO:0097367 | 33 | 1 | 0.005417 |
| nucleic acid binding | GO:0003676 | 36 | 1 | 0.005744 |
| anion binding | GO:0043168 | 38 | 1 | 0.005880 |
| metal ion binding | GO:0046872 | 41 | 1 | 0.005880 |
| cation binding | GO:0043169 | 42 | 1 | 0.005880 |
| nucleotide binding | GO:0000166 | 43 | 1 | 0.005880 |
| small molecule binding | GO:0036094 | 43 | 1 | 0.005880 |
| nucleoside phosphate binding | GO:1901265 | 43 | 1 | 0.005880 |
| ion binding | GO:0043167 | 65 | 1 | 0.008676 |
| organic cyclic compound binding | GO:0097159 | 76 | 1 | 0.009690 |
| heterocyclic compound binding | GO:1901363 | 76 | 1 | 0.009690 |
| binding | GO:0005488 | 108 | 1 | 0.013457 |
| **Biological Process** | | | | |
| **Term description** | **Term identity** | **Proteins** | **Hits** | ***P-value_fdr*** |
| regulation of biological quality | GO:0065008 | 10 | 2 | 7.29E-05 |
| biological_process | GO:0008150 | 194 | 3 | 7.29E-05 |
| single-organism metabolic process | GO:0044710 | 50 | 2 | 0.000599 |
| single-organism cellular process | GO:0044763 | 83 | 2 | 0.000599 |
| organic substance biosynthetic process | GO:1901576 | 97 | 2 | 0.000599 |
| cellular biosynthetic process | GO:0044249 | 98 | 2 | 0.000599 |
| single-organism process | GO:0044699 | 100 | 2 | 0.000599 |
| biosynthetic process | GO:0009058 | 105 | 2 | 0.000599 |
| regulation of DNA recombination | GO:0000018 | 1 | 1 | 0.000599 |
| telomere maintenance | GO:0000723 | 1 | 1 | 0.000599 |
| regulation of DNA repair | GO:0006282 | 1 | 1 | 0.000599 |
| sesquiterpenoid metabolic process | GO:0006714 | 1 | 1 | 0.000599 |
| juvenile hormone metabolic process | GO:0006716 | 1 | 1 | 0.000599 |
| juvenile hormone biosynthetic process | GO:0006718 | 1 | 1 | 0.000599 |
| terpenoid metabolic process | GO:0006721 | 1 | 1 | 0.000599 |
| regulation of double-strand break repair via homologous recombination | GO:0010569 | 1 | 1 | 0.000599 |
| sesquiterpenoid biosynthetic process | GO:0016106 | 1 | 1 | 0.000599 |
| terpenoid biosynthetic process | GO:0016114 | 1 | 1 | 0.000599 |
| telomere organization | GO:0032200 | 1 | 1 | 0.000599 |
| cellular hormone metabolic process | GO:0034754 | 1 | 1 | 0.000599 |
| regulation of DNA metabolic process | GO:0051052 | 1 | 1 | 0.000599 |
| anatomical structure homeostasis | GO:0060249 | 1 | 1 | 0.000599 |
| regulation of response to stress | GO:0080134 | 1 | 1 | 0.000599 |
| regulation of cellular response to stress | GO:0080135 | 1 | 1 | 0.000599 |
| regulation of double-strand break repair | GO:2000779 | 1 | 1 | 0.000599 |
| regulation of response to DNA damage stimulus | GO:2001020 | 1 | 1 | 0.000599 |
| primary metabolic process | GO:0044238 | 136 | 2 | 0.000622 |
| organic substance metabolic process | GO:0071704 | 136 | 2 | 0.000622 |
| cellular metabolic process | GO:0044237 | 137 | 2 | 0.000622 |
| cellular process | GO:0009987 | 170 | 2 | 0.000841 |
| DNA replication | GO:0006260 | 2 | 1 | 0.000841 |
| isoprenoid metabolic process | GO:0006720 | 2 | 1 | 0.000841 |
| isoprenoid biosynthetic process | GO:0008299 | 2 | 1 | 0.000841 |
| regulation of hormone levels | GO:0010817 | 2 | 1 | 0.000841 |
| hormone metabolic process | GO:0042445 | 2 | 1 | 0.000841 |
| hormone biosynthetic process | GO:0042446 | 2 | 1 | 0.000841 |
| homeostatic process | GO:0042592 | 2 | 1 | 0.000841 |
| DNA geometric change | GO:0032392 | 3 | 1 | 0.001167 |
| DNA duplex unwinding | GO:0032508 | 3 | 1 | 0.001167 |
| DNA conformation change | GO:0071103 | 3 | 1 | 0.001167 |
| DNA recombination | GO:0006310 | 4 | 1 | 0.001383 |
| lipid metabolic process | GO:0006629 | 4 | 1 | 0.001383 |
| lipid biosynthetic process | GO:0008610 | 4 | 1 | 0.001383 |
| cellular lipid metabolic process | GO:0044255 | 4 | 1 | 0.001383 |
| regulation of response to stimulus | GO:0048583 | 4 | 1 | 0.001383 |
| DNA metabolic process | GO:0006259 | 7 | 1 | 0.002223 |
| DNA repair | GO:0006281 | 7 | 1 | 0.002223 |
| cellular response to DNA damage stimulus | GO:0006974 | 7 | 1 | 0.002223 |
| cellular response to stress | GO:0033554 | 7 | 1 | 0.002223 |
| chromosome organization | GO:0051276 | 11 | 1 | 0.003422 |
| response to stress | GO:0006950 | 12 | 1 | 0.003660 |
| oxidation-reduction process | GO:0055114 | 13 | 1 | 0.003888 |
| single-organism organelle organization | GO:1902589 | 17 | 1 | 0.004987 |
| single-organism biosynthetic process | GO:0044711 | 21 | 1 | 0.005935 |
| cellular response to stimulus | GO:0051716 | 21 | 1 | 0.005935 |
| transport | GO:0006810 | 23 | 1 | 0.006164 |
| organelle organization | GO:0006996 | 23 | 1 | 0.006164 |
| establishment of localization | GO:0051234 | 23 | 1 | 0.006164 |
| localization | GO:0051179 | 25 | 1 | 0.006585 |
| response to stimulus | GO:0050896 | 30 | 1 | 0.007768 |
| regulation of nucleobase-containing compound metabolic process | GO:0019219 | 41 | 1 | 0.010435 |
| cellular component organization | GO:0016043 | 51 | 1 | 0.012763 |
| cellular component organization or biogenesis | GO:0071840 | 54 | 1 | 0.013297 |
| regulation of nitrogen compound metabolic process | GO:0051171 | 57 | 1 | 0.013814 |
| regulation of cellular metabolic process | GO:0031323 | 60 | 1 | 0.014097 |
| regulation of primary metabolic process | GO:0080090 | 60 | 1 | 0.014097 |
| nucleic acid metabolic process | GO:0090304 | 63 | 1 | 0.014579 |
| regulation of macromolecule metabolic process | GO:0060255 | 65 | 1 | 0.014818 |
| regulation of metabolic process | GO:0019222 | 66 | 1 | 0.014827 |
| regulation of cellular process | GO:0050794 | 75 | 1 | 0.016327 |
| macromolecule biosynthetic process | GO:0009059 | 76 | 1 | 0.016327 |
| cellular macromolecule biosynthetic process | GO:0034645 | 76 | 1 | 0.016327 |
| nucleobase-containing compound metabolic process | GO:0006139 | 77 | 1 | 0.016327 |
| cellular aromatic compound metabolic process | GO:0006725 | 78 | 1 | 0.016327 |
| regulation of biological process | GO:0050789 | 81 | 1 | 0.016675 |
| biological regulation | GO:0065007 | 82 | 1 | 0.016675 |
| heterocycle metabolic process | GO:0046483 | 84 | 1 | 0.016675 |
| organic cyclic compound metabolic process | GO:1901360 | 84 | 1 | 0.016675 |
| cellular macromolecule metabolic process | GO:0044260 | 110 | 1 | 0.021448 |
| cellular nitrogen compound metabolic process | GO:0034641 | 111 | 1 | 0.021448 |
| macromolecule metabolic process | GO:0043170 | 115 | 1 | 0.021941 |
| nitrogen compound metabolic process | GO:0006807 | 117 | 1 | 0.022047 |
| metabolic process | GO:0008152 | 160 | 1 | 0.029707 |
| **Cellular Component** | | | | |
| **Term description** | **Term identity** | **Proteins** | **Hits** | ***P-value_fdr*** |
| membrane-bounded organelle | GO:0043227 | 95 | 2 | 0.000227 |
| intracellular membrane-bounded organelle | GO:0043231 | 95 | 2 | 0.000227 |
| intracellular organelle | GO:0043229 | 117 | 2 | 0.000227 |
| organelle | GO:0043226 | 118 | 2 | 0.000227 |
| intracellular part | GO:0044424 | 156 | 2 | 0.000227 |
| intracellular | GO:0005622 | 158 | 2 | 0.000227 |
| cell part | GO:0044464 | 165 | 2 | 0.000227 |
| cell | GO:0005623 | 166 | 2 | 0.000227 |
| mitochondrion | GO:0005739 | 23 | 1 | 0.004634 |
| integral component of membrane | GO:0016021 | 24 | 1 | 0.004634 |
| intrinsic component of membrane | GO:0031224 | 24 | 1 | 0.004634 |
| membrane part | GO:0044425 | 29 | 1 | 0.005132 |
| membrane | GO:0016020 | 35 | 1 | 0.005716 |
| nucleus | GO:0005634 | 67 | 1 | 0.010150 |
| cytoplasmic part | GO:0044444 | 76 | 1 | 0.010743 |
| cytoplasm | GO:0005737 | 105 | 1 | 0.013902 |
| cellular_component | GO:0005575 | 195 | 1 | 0.024230 |
