# Supplementary material for: Characterization of the transcriptional cellular response in midgut tissue of temephos-resistant Aedes aegypti larvae
Source: Parasit Vectors. 2025 May 14;18:174. doi: 10.1186/s13071-025-06675-5 (PMC12076995; doi:10.1186/s13071-025-06675-5)
Supplement: Supplementary file 1 — Supplementary Material 1 [file 13071_2025_6675_MOESM1_ESM.zip › Supplementary table 4 Helvecio et al V9.docx]

**Supplementary table 4.** Gene Ontology of enriched terms downregulated in the RecR colony.

| **Molecular Function** | | | | |
| --- | --- | --- | --- | --- |
| **Term description** | **Term identity** | **Proteins** | **Hits** | ***P-value_fdr*** |
| hydrolase activity. acting on ester bonds | GO:0016788 | 6 | 1 | 0.002813 |
| zinc ion binding | GO:0008270 | 9 | 1 | 0.002813 |
| transition metal ion binding | GO:0046914 | 14 | 1 | 0.002917 |
| hydrolase activity | GO:0016787 | 26 | 1 | 0.004063 |
| metal ion binding | GO:0046872 | 41 | 1 | 0.004376 |
| cation binding | GO:0043169 | 42 | 1 | 0.004376 |
| ion binding | GO:0043167 | 65 | 1 | 0.005804 |
| catalytic activity | GO:0003824 | 78 | 1 | 0.006095 |
| binding | GO:0005488 | 108 | 1 | 0.007501 |
| molecular_function | GO:0003674 | 164 | 1 | 0.010251 |
| **Biological Process** | | | | |
| **Term description** | **Term identity** | **Proteins** | **Hits** | ***P-value_fdr*** |
| catabolic process | GO:0009056 | 12 | 1 | 0.00225 |
| metabolic process | GO:0008152 | 160 | 1 | 0.01213 |
| biological_process | GO:0008150 | 194 | 1 | 0.01213 |
